# An Image Schematic Analysis of Conceptual Metaphors of Adolescents’ Lived Experiences of Depression

**DOI:** 10.31083/AP47293

**Published:** 2025-10-13

**Authors:** Yurou Liang, Yu Deng

**Affiliations:** ^1^School of English Studies, Sichuan International Studies University, 400031 Chongqing, China; ^2^College of Language Intelligence, Sichuan International Studies University, 400031 Chongqing, China

**Keywords:** depression, adolescents, conceptual metaphor, image schema

## Abstract

**Background:**

Depression has a substantial impact on adolescents’ mental health. This study investigated adolescents’ use of conceptual metaphors to convey their experiences with depression and analyzed the role of image schemas in structuring their metaphorical conceptualization of the condition. The objective was to elucidate the cognitive mechanisms underlying the framing of depression.

**Methods:**

Based on the metaphorical expressions from interview data collected from 20 adolescents (aged 15 to 19) diagnosed with depression, this study conducted conceptual metaphor and image schema analyses of narrative discourse related to depression.

**Results:**

The analyses revealed that CONTAINER, THING, PHYSICAL ENTITY, and LOCATION metaphors were primarily used to conceptualize the disorder itself, life with depression, communication and personal environment, medication and therapy, as well as moods, thoughts, and emotions. These metaphors illuminated participants’ concerns and challenges in their lived experiences with depression. Furthermore, image schemas such as CONTAINER, PATH, COMPULSION, ENABLEMENT, and VERTICALITY were frequently employed as subjects metaphorically reasoned about their experiences with depression.

**Conclusions:**

The analyses of conceptual metaphors and image schemas in narrative discourse revealed that adolescents tend to draw on bodily experiences to metaphorically interpret their lived experiences of depression. Mental health professionals may benefit from paying closer attention to the image schemas and metaphorical expressions used by patients with depression when assessing their mental health condition.

## Main Points

1. We explored narratives of adolescents with depression to reveal how they employed metaphors to communicate their experiences of depression and how image schemas functioned in their metaphorical thinking about depression.

2. Individuals with depression mainly used CONTAINER, THING, PHYSICAL ENTITY, and LOCATION metaphors to conceptualize the disorder itself, life with depression, communication and personal environment, medication and therapy, and moods, thoughts, and emotions. CONTAINER, PATH, COMPULSION, ENABLEMENT, and VERTICALITY image schemas were frequently used when they were metaphorically reasoning about their experiences of depression.

3. Adolescents tended to employ their bodily experiences to metaphorically interpret their lived experiences of depression.

4. Mental health professionals could pay closer attention to image schemas and metaphorical expressions used by patients with depression when evaluating their mental health condition.

## 1. Introduction

Upwards of 50% of adults with mental health conditions experience psychological disturbances during adolescence [1], making it crucial to implement timely and effective interventions to reduce the long term burden of illness [2, 3]. More than 700,000 people worldwide die by suicide each year due to depression-related causes, and approximately 280 million people live with depression [4]. With a high morbidity rate, depression has a profound impact on adolescent mental health. Given the extensive negative impact of depression on the well-being of diagnosed patients, it is essential for healthcare personnel to conduct early screening for this disorder.

When individuals discuss or write about their experiences with depression, they may use a variety of metaphors to express their lived reality [5]. Metaphors, as a mode of thought, can reveal the underlying conceptual and cognitive processes [6], and serve as valuable tool for mental health professionals in assessing mental health conditions. Conceptual metaphors have been widely applied to understand experiences of intense emotions and illness due to their role in shaping human cognition and mental imagery [7, 8]. More broadly, metaphors can reveal the conceptualization of complex, abstract, or intangible ideas, concepts, or emotions through embodied schemas and gestalts.

The need for context-specific understanding of adolescent mental health experiences has been recognized as a critical gap in global research [9]. Metaphor serves as a powerful linguistic and cognitive tool that young people rely on to articulate their understanding and experiences with depression. Through relatable and vivid imagery, adolescents convey the intricacies of their depression experiences, constructing meaningful narratives that encapsulate their personal struggles and emotions [10].

Adolescents’ metaphors for depression are notably distinctive. Compared to adults, adolescents are in an earlier stage of cognitive, emotional, and social development. As a result, they encounter unique challenges, including academic pressures and failures [11], interpersonal struggles, school transitions, bullying, and self-harm risks. The thematic content of metaphors used by adolescents with depression reflects context-specific particularities, shaped by sociocultural and developmental factors. Beyond the mental condition itself, their metaphors often focus on life challenges when affected by depression, such as school work, interpersonal relationships, and experiences of bullying. In general, metaphors can help adolescents process and communicate their struggles, allowing them to share their illness narratives in a way that fosters understanding and connection with others [12].

Image schema is the recurring, dynamic pattern of human perceptual interactions and motor programs that gives coherence and structure to our experience [8]. For instance, VERTICALITY schema is abstracted from the “verticality” experiences, images, and perceptions, such as climbing stairs and measuring the heights of objects. Image schema is extensively spotted in metaphor analysis of emotional states and thinking processes [13]. Essentially, image schemas can reflect people’s concrete experiential patterns and thinking mechanism underlying metaphors.

Building on this foundation, this study drew upon Conceptual Metaphor Theory and Image Schema Theory to explore the narratives of adolescents with depression. Given the complexity of mental illness experiences, a qualitative approach was adopted to analyze the metaphors used by patients and their underlying image schemas.

Metaphor analysis has been widely utilized in depression-related discourse studies. Charteris-Black identified metaphors in interview data from individuals who had experienced depression. The findings revealed that containment and constraint metaphors were frequently associated used by patients to express depression, and that metaphorical thinking could facilitate recovery [14]. Yu and Tay [15] addressed image schematic metaphors related to three themes in therapeutic discourse, namely, anger, anxiety, and depression, identified in an online archive. They uncovered the potential relationship between types of image-schematic metaphors and distinct topical themes of therapeutic discourse, arguing that metaphors have diagnostic implications [15]. Tonon [16] analyzed metaphors from online forums and found that depression influences individuals at both the physical and psychological levels. Furthermore, language becomes symptomatic of people’s internal bodily and mental states, reflecting their emotional distress and feelings of depression. This study supports the idea that metaphors can help diagnose and treat depression [16]. Coll-Florit *et al*. [17] conducted a metaphor analysis of depression based on a corpus of blogs written by people with major depressive disorder. Metaphors, such as “psychiatrists were conceptualized as captors” and “prejudice as wright and force” were identified. Furthermore, metaphors relating to loss of control were motivated by BALANCE image schema [17]. Shi and Khoo [4], who constructed a Chinese corpus from an online depression community, found that dominant metaphors in other languages, such as DESCENT, JOURNEY, and IMBALANCE, were also present among Chinese patients. Notably, they identified new metaphors, such as THEATRICAL ROLE and CYBERCULTURE, which reflected characteristics of the Chinese sociocultural environment, personal embodiment, and idiosyncrasies [4].

As a subtype of depression, postpartum depression has received extensive attention in metaphor studies. Drawing on metaphorical expressions used by women with postpartum depression, Beck [18] demonstrated that metaphors could serve as an important means for mothers to articulate their experiences of interacting with their infants. Moreover, metaphors help healthcare providers assess whether a woman is struggling with postpartum depression, enabling timely and proper intervention [18]. Beck [19] further argued that metaphors not only provide a new voice for individuals experiencing postpartum depression but also offer valuable insights for healthcare providers. These insights aid in identifying women at risk and developing targeted interventions [19]. Al-Jumaili *et al*. [20] examined metaphorical expressions in Elif Shafak’s novel and found that Shafak represented the abstract state of postpartum depression through physical and material experiences via cross-domain mapping process. Their analysis highlights how metaphors facilitate the communication of complex and abstract states related to depression by grounding them in physical experiences [20].

Depression metaphors can also be expressed through non-verbal behaviors. Fahlenbrach [21] found that metaphors conveyed in the audiovisual representation of emotions can relate an emotion with a different domain in filmmaking. The metaphor analysis of depression in short, wordless animated films revealed cross-domain patterns such as DEPRESSION IS A DARK MONSTER and DEPRESSION IS AS DARK CONFINING SPACE. This suggests that conceptual metaphors that may not be easily articulated through language can instead be conveyed visually through animation [22].

Shifting the focus to young adults, Roystonn *et al*. [10] collected data from semi-structured interviews to investigate how patients make sense of their experiences with depression through metaphors. The results confirmed that young adults frequently used metaphors to describe their experiences of depression, and that metaphorical language could provide important implications for clinical practice and healthcare settings [10].

Taken together, existing studies demonstrate that individuals, when grappling with depressive sentiments that are recalcitrant to direct verbalization, frequently turn to metaphorical constructs. Metaphors serve as a linguistic bridge, spanning the divide between the articulable aspects of their emotional state and the ineffable experiences that reside within their cognitive landscape [23, 24]. Metaphor and image schema emerge as valuable tools in the identification of depression and in helping healthcare professionals to communicate and understand patients’ lived experiences of depression.

Despite previous studies underscoring the role of metaphor in discussions of depression, scant attention has been directed towards examining the contexts within which young people employ metaphors to frame their lived experience of depression. Furthermore, there exists a dearth of research elucidating the mechanisms by which domain mappings are realized within metaphorical constructs pertaining to depression. It should be noted that the recurrent patterns of image schema could provide a foundation for the source domains of metaphors [15]. Hence, this study aimed to explore the conceptual metaphors used by adolescents in describing their experiences of depression, in order to reveal their emotional states, concerns, and problems. Specifically, this study intended to analyze the themes of the metaphors adolescents used and investigate how they conveyed their emotional or physical concerns through these metaphors. The study sought to provide a comprehensive depiction of the challenges faced by adolescent patients, thereby shedding light on the multifaceted nature of their experiences with depression. Moreover, this study was designed to interpret the image schemas that underpin the conceptual metaphors, thereby revealing the concrete experiential patterns and cognitive mechanisms involved in the presentation and construction of the lived experiences of people with depression. By scrutinizing the manner in which conceptual metaphors were articulated and how the cross-domain mappings of metaphors were instantiated through the experiential structures of image schemas, this study endeavored to elucidate the cognitive mechanisms at work in adolescents with depression. Gaining such insights could promote the identification of mental health challenges and support coping strategies.

## 2. Material and Methods

### 2.1 Research Subjects

All the interview data used in this study were collected from the Healthtalk website (https://healthtalk.org/), an existing open-access database that archives interviews with individuals experiencing various health conditions along with their profile information. The database categorizes interviews alphabetically by health condition topics. Given the research gap identified in this paper, only interviews with adolescents diagnosed with depression (aged 15 to 19 years) were selected, resulting in a dataset of 20 narratives. Thus, this study included 20 research participants, comprising 15 females and 5 males, from the “Depression and Low Mood (Young People)” section on Healthtalk. These participants had experienced depression and low mood and shared their lived experiences during the interviews. The transcripts of these interviews, totaling 37,972 words, were collected for analysis. During the interviews, participants discussed various aspects of their lives with depression, including childhood experiences, self-harm, suicidal thoughts, treatments for depression, and interpersonal relationships.

It should be noted that all analyses in this study were conducted in full compliance with Healthtalk’s terms of use and privacy policies to ensure the confidentiality and security of participant information.

### 2.2 Study Design

A qualitative research design was adopted in this study. The selection and identification of conceptual metaphors used by research subjects were conducted, followed by the labeling of underlying image schemas. Once all coding and labeling were completed, the interpretation of metaphors and image schemas relating to expression was carried out in accordance the literature.

### 2.3 Data Analysis

#### 2.3.1 Metaphor Analysis

To gain an in-depth understanding of the mental health challenges faced by adolescents diagnosed with depression, this study analyzed different types of metaphors regarding their lived experiences of depression. Drawing on the metaphor detection method of Coll-Florit and Climent [25], we coded the metaphors in four steps:

1. Manual pre-selection of candidate metaphorical expressions.
2. Use of Metaphor Identification Procedure (MIP) to identify the metaphorical focus [26], which involves four sub-steps: (1) generated a general understanding of the texts, that is to make clear the background information, main information, and topic of the text; (2) manually identified lexical units, dividing texts into one-word units, compound-word units, or phrasal-verb units that are potentially metaphorical in context; (3) compared each lexical unit’s contextual meaning with its basic meaning, referencing the online Macmillan Dictionary. In this step, the contextual and basic meanings of each lexical unit were considered separately and then compared to determine whether the contextual meaning contrasts with the basic meaning but remains comprehensible through comparison;
3. Use of metaphor compendia for labeling metaphor domains;
4. If no suitable models were found in these compendia, labeling strategies [25] were adopted to annotate the domains of candidate metaphors.

It is noteworthy that metaphors were coded with reference to a compendium of depression-related metaphors compiled from the literature reviewed in the Introduction, the Master Metaphor List [27], and metaphor thematic fields [28, 29].

**Fig. 1.**
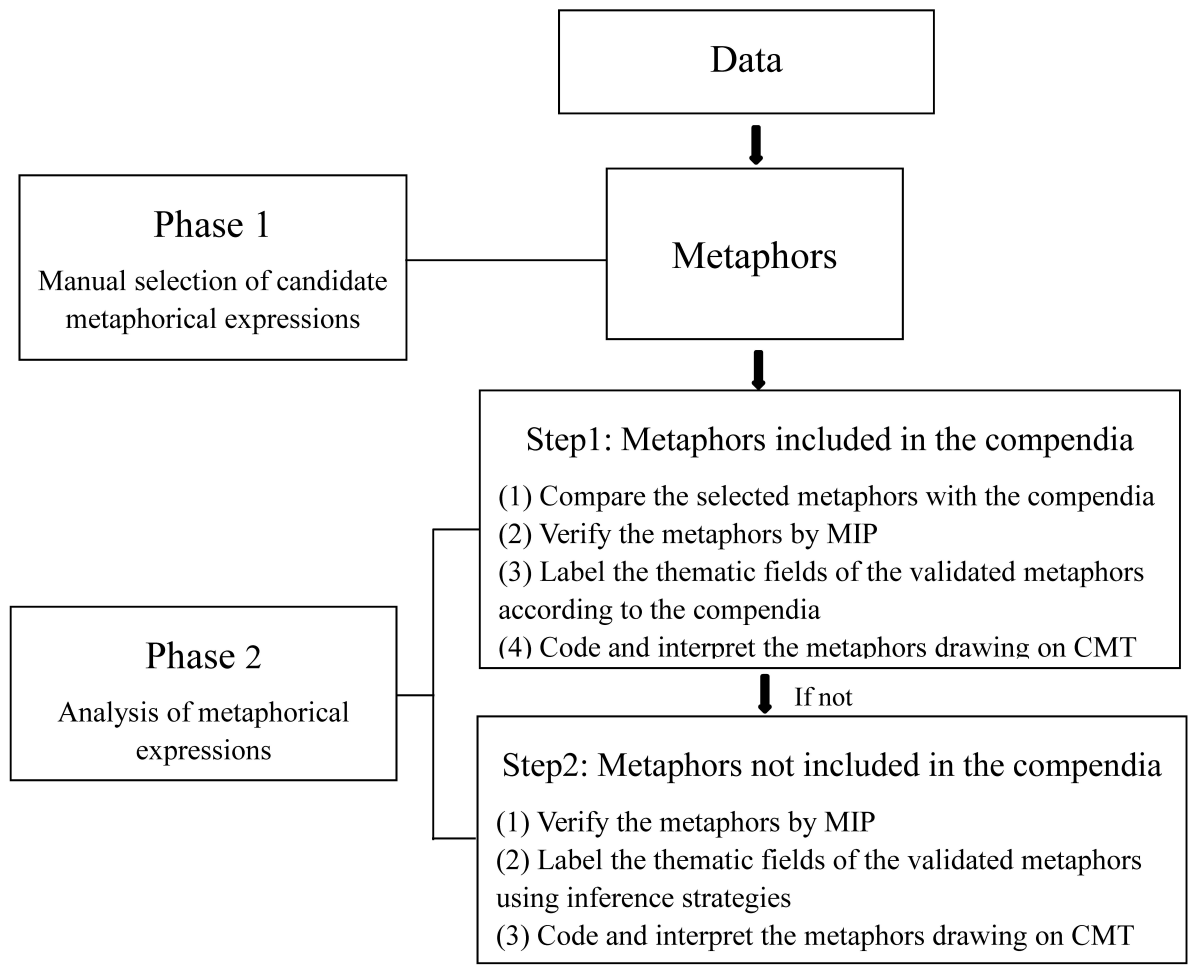
**The overall procedure for metaphor analysis**. Phase 1 indicates that the authors label the candidate metaphorical expressions by referring to the online Macmillan Dictionary. Phase 2 is the verification or rejection of the candidate metaphors, and only the verified ones are reserved. If a candidate metaphor matches with one in the compendia, it is analyzed following the process in step 1. If not, it is processed according to step 2. MIP, Metaphor Identification Procedure; CMT, Conceptual Metaphor Theory.

The metaphor analysis method involved two main phases, as shown in Fig. 1.

#### 2.3.2 Image Schema Analysis

The coding of image schemas in participants’ narratives drew on Johnson [8] and other published literature [13, 15, 30, 31]. Table 1 lists the most common image schemas proposed by Johnson [8], while Table 2 presents exemplar metaphors representing the 25 distinct types of image schemas identified in this study. A detailed description and interpretation were provided for each image schema as it first emerged in the analyses. It is possible that a conceptual metaphor incorporates a “compound” of different image schematic groundings [32], as the study of image schemas underlying metaphors in the communication of depression focused not only on the linguistic features of expressions but also on therapeutically relevant factors such as emotions and thoughts. Thus, in this study, the onefold image schema that best represented the speaker’s specific expressive needs was selected for metaphor analysis [13].

**Table 1.** **List of image schemas**.

CONTAINER	BALANCE	COMPULSION
BLOCKAGE	COUNTERFORCE	RESTRAINT REMOVAL
ENABLEMENT	ATTRACTION	MASS-COUNT
PATH	LINK	CENTER-PERIPHERAL
CYCLE	NEAR-FAR	SCALE
PART-WHOLE	MERGING	SPLITTING
FULL-EMPTY	MATCHING	SUPER IMPOSITION
ITERATION	CONTACT	PROCESS
SURFACE	OBJECT	COLLECTION

**Table 2.** **Metaphor examples for each image schema identified in this study**.

CONTAINER	I felt like I was at the bottom of **a deep well**.
BLOCKAGE	The smallest things that seem so simple like having a bath or a shower, became **mountains** for me.
ENABLEMENT	I don’t know that I’m doing it, I don’t know **how to turn it on and off**.
PATH	The only problem was the main focus of each session seemed to be about** getting me back into** school, not the underlying issues.
CYCLE	But I just got into this **cycle**, I do not like men at all.
PART-WHOLE	I have had this for such a long time I have had the **headaches side** of it, I have had the **psychosomatic side** of depression.
FULL-EMPTY	Now draw a line and show us **how full** your bad mood is today.
SURFACE	Like just** touching on the surface**, it’s getting to the point we’re getting deeper and deeper about it.
BALANCE	Because in the end, what depression is a **chemical imbalance**, and all the anti-depressants do is balance the chemical back up.
COUNTERFORCE	Depression is a** battle**, one that at the time you never feel like you’re going to win.
ATTRACTION	I mean the self-harm thing went on for a good couple of years and it’s, you know, like smoking it’s an **addiction**.
LINK	Every so often I go into hospital and that made me feel even worse because I was **cut off from** everybody else.
NEAR-FAR	I guess I really didn’t want to be sort of like seen as **anyone just in the corner**.
MATCHING	Because I guess I knew because we are all this **same kind of thing**, all there for the same kind of reason.
CONTACT	He’s just a very unpleasant **character** to **live with**.
OBJECT	I don’t like to get too intimately attached cos then it’s just gonna **get broken** at some point.
COMPULSION	Because sometimes my panic attacks just **jump out of nowhere**, and if it happened like that, then not a chance.
RESTRAINT REMOVAL	It’s **getting over that hurdle** of trusting them is the hardest thing with the relationship you just break down.
CENTER-PERIPHERAL	I’m planning to move out and get my own place next year, so that’s kind of a big** milestone**, trying to get my driving license.
SCALE	With the help of my brilliant counsellor slowly my confidence **built up**.
SPLITTING	I just I want people to know that I’m** two different people**.
SUPER IMPOSITION	To them I wasn’t a **victim** I was just Gemma and that made me realise that too.
PROCESS	But if someone’s honestly** having such a difficult time** that’s the only way they feel they can let out how they’re feeling, then they need help.
COLLECTION	I’ve had** loads of support** from my mum, grandparents, and uncle.
VERTICALITY	It got to a stage where I was just really** down**.

The metaphorical focus of the exemplar metaphors is boldfaced, representing the 25 distinct types of image schemas identified in this study.

In this and subsequent cited examples in the following sections, the metaphorical focus is boldfaced to indicate the verified metaphorical expressions.

#### 2.3.3 Analytical Procedure

The specific metaphor and image schema analysis is illustrated by the following example.

Text: I’d say probably the media was the main thing that sort of, like all the factors probably led up to maybe me more vulnerable to depression, but the one that sort of **triggered** it and **set it off** was the media.

Metaphor Analysis

(1) Candidate metaphorical expressions: triggered; set it off.

(2) No match found in the compendia.

(3) Using MIP to verify the candidate metaphorical expressions:

Validation: “Triggered” and “set it off”: the onset of depression.

(4) Thematic field: DEPRESSION.

(5) Conceptual metaphor: DEPRESSION IS PHYSICAL ENTITY.

Metaphor domains: Target domain: depression; Source domain: bomb.

Image Schema Analysis.

(1) Image schema: COMPULSION, which involves an external force with a given magnitude, trajectory, and direction [8].

(2) COMPULSION is a structure that generally includes the following elements: a force with a certain direction that is either irresistible or can be modified; an object that is affected or potentially affected by the force, moving in the same direction as the force; a potential trajectory or path of motion along which the object moves. In some cases, the structure includes a goal or destination toward which the force or the affected object moves [8]. This schema highlights our experience of force, the involuntary activity guided by the force, and the uncontrollable result of the forced activity. The depression metaphor in this case reflects the speaker’s perception of a metaphorical force acting upon, leading to an inevitable outcome, a pattern that aligns precisely with the COMPULSION schema. This schema emphasizes the involuntary and uncontrollable nature of the symptom [33]. In this case, the media is conceptualized as an irresistible external force acting on the speaker, ultimately leading to depression.

To maximize coding reliability and mitigate the influence of subjective bias, the analyses were conducted by three independent coders: two postgraduate students specializing in metaphor and one professional linguist with a PhD. To ensure coding accuracy and consistency, the three coders first independently coded the metaphors and image schemas in the initial round of analysis. They then met to discuss and resolve disagreements in a second round. In cases where two coders assigned divergent labels, the matter was referred to the third coder for further evaluation. The coding of a case was considered valid only when consensus or majority agreement was reached among the coders. In the first round of coding, 235 out of 278 metaphor annotations (84.53%) and 257 out of 278 image schemas (92.44%) reached a consensus among the three coders. In the second round, all remaining disagreements were resolved through discussion among the coders.

## 3. Results

A total of 267 metaphorical expressions and 25 types of image schema were identified in the interview data. The metaphors were classified into 6 categories: “depression”, “communication” and “personal environment”, “moods”, “thoughts and emotions”, “medicine and therapy”, “habits and interests”, and “others”. The most frequently used image schemas within metaphorical expressions included the schema of CONTAINER, ENABLEMENT, PATH, VERTICALITY, COMPULSION, and BLOCKAGE. As shown in Table 3, 40.82% of the metaphorical expressions were associated with depression. The image schemas most frequently used by patients to construct metaphors were CONTAINER, PATH, ENABLEMENT, and COMPULSION.

**Table 3.** **Main metaphor categories and image schemas identified and their quantities**.

Metaphor categories	Instances	Percentage	Image schemas
Depression	109	40.82%	CONTAINER(22)
			PATH (17)
			ENABLEMENT (10)
			COMPULSION(7)
Disorder	53	48.62%	PATH (10)
			CONTAINER(8)
			ENABLEMENT(5)
People with depression	29	26.61%	CONTAINER(10)
			LINK(6)
			SPLITTING(5)
Life with depression	15	13.76%	CONTAINER(3)
			PATH (3)
			VERTICALITY(2)
School and college work	12	11.01%	CYCLE(2)
			PATH (2)
			PART-WHOLE(2)
Moods, thoughts and emotions	50	18.73%	VERTICALITY(15)
			COMPULSION (4)
			FULL-EMPTY (4)
Communication and personal environment	44	16.48%	CONTAINER(8)
			ENABLEMENT(8)
			COMPULSION(6)
Medicine and therapy	31	11.61%	CONTAINER(5)
			COMPULSION(4)
			PROCESS(3)
Other	23	8.61%	ENABLEMENT(6)
			OBJECT(4)
			PATH (3)
Habit and interests	10	3.75%	PATH (6)
			CONTAINER(2)
Total	267	100%	25 Types

Metaphors of moods, thoughts and emotions accounted for 18.73%, referring to participants’ experiences of moods, emotions, and thoughts. The image schema of VERTICALITY, COMPULSION, and FULL-EMPTY were used in constructing patients’ experience of depressed emotions and feelings. Metaphors related to communication and personal environment and metaphors concerning medicine and therapy accounted for 16.48% and 11.61%, respectively. These metaphors reflected how participants understood interpersonal relationships, self-perception, and social surroundings, as well as their experiences with medication, counseling, and treatment. Notably, these two types of metaphors shared CONTAINER and COMPULSION image schema. Furthermore, ENABLEMENT schema was used for metaphors relating to communication and personal environment, while PROCESS schema was employed in metaphors concerning medicine and therapy. Metaphors related to habits and interests comprised 3.75% of the data. Participants described their daily activities and personal interests using LOCATION, CONTAINER, and ABSTRACTION metaphors. To structure these experiences, they applied the LINK and CONTAINER schemas in the mapping process between target and source domains. Finally, metaphors concerning the mind and brain, self-harm, and bullying were classified as “other metaphors” due to their low frequency. In these metaphors, participants conceptualized their experiences using image schemas such as ENABLEMENT, OBJECT, and PATH in the metaphorical conceptualization process.

### 3.1 Image Schematic Analysis of Depression Metaphors

#### 3.1.1 Depression 

Metaphors related to depression accounted for the largest proportion, encompassing not only the disorder itself but also the individuals experiencing it, as well as their daily lives and academic work. As shown in Table 3, 48.62% of the metaphorical expressions were associated with the disorder. Secondly, 26.61% of the metaphorical expressions referred to individuals experiencing depression, who were primarily conceptualized as possessing an alternate identity, as containers, or as machines. Metaphors related to life with depression accounted for 13.76% of the metaphorical expressions, relating to various physical entities. Meanwhile, 11.01% of the metaphorical expressions represented school and college work, which was mainly conceptualized as different objects.

The source domains of depression metaphors were primarily associated with physical entities, living organisms, and locations. Through conceptual metaphors, participants tended to conceptualize depression as THING, PHYSICAL ENTITY, CONTAINER, SPATIAL MOTION, LOCATION, LIVING ORGANISM, CYCLE, JOURNEY, and BATTLE. THING and PHYSICAL ENTITY metaphors were prominent in participants’ narratives, where depression was conceptualized as both general things and physical object respectively, such as a bomb, a building, a fluid, or a machine. These metaphors reflected the diverse ways in which participants perceived depression. For instance, the onset of depression was likened to a bomb explosion, suggesting that the disorder is triggered by one or multiple factors.

Participants employed multiple image schemas to structure their experiences of depression. For instance, (1a) illustrates BALANCE image schema, which involves a symmetrical or proportional arrangement of forces around a point or axis, where “balance” is maintained. BALANCE schema was discussed from multiple perspectives from various perspectives in Johnson’s work [8]. According to Johnson, “We tend to seek temporary homeostasis where we are emotionally balanced, stable. The ideal is a balanced personality. This requires that sufficient weight be given to each of the parts or dimensions of our character”. In this example, the concept of physical balance is central. The participant conceptualized depression as a chemical imbalance, which could be corrected by restoring equilibrium through antidepressants. Specifically, health was depicted as the balanced distribution of chemical components. Here, both health and depression were understood in terms of quantity and weight: when this balance was disrupted, depression emerged in the participant’s body. Here, the FORCE-related image schema COMPULSION involves an external force with a given magnitude, a trajectory, and a direction [8]. The depression metaphors in (1b) were derived from participants’ concrete experience of compulsive force acted on the target, which constituted the recurrent patterns in COMPULSION schema. This schema emphasizes the involuntary and uncontrollable nature of the symptom [33]. Furthermore, external factors such as media influence and a lack of confidence were conceptualized as two irresistible forces acting upon participants, ultimately leading to the onset of depression. Conceptual metaphors of depression were also structured around the source domain of places and bounded spaces, as exemplified in DEPRESSION IS LOCATION and DEPRESSION IS CONTAINER.

SPATIAL MOTION and LOCATION metaphors were mainly framed through the embodied experiential patterns of PATH schema, featured with routes for a subject moving from the starting point to the endpoint. In example (2a), experiencing depression was conceptualized as the unwillingly taken route that the participant was taking. Here, fitness was understood as the starting point, while depression, framed as a downward spatial motion, represented the goal of the path. This type of metaphor was also organized by BLOCKAGE schema (2b), where a force vector encounters a barrier and is subsequently redirected in multiple possible directions [8]. In this example, efforts to study depression were interpreted as the force vector, while hardships, difficulties, and other limiting factors represented the obstacle in the BLOCKAGE schema. Such obstacle obstructed researchers’ ability to “see” depression, hindering their capacity to explore and develop a deeper understanding of the disorder.

This study also identified numerous metaphors related to containment and constraint. CONTAINER metaphors were the typical manifestation of CONTAINER image schema, accounting for the experience and cognition of “boundedness” and “out”. Depression was construed as a restrictive container, confining individuals within its limits, rendering them inaccessible to others (3a), and leading them to yearn for liberation (3b).

Other metaphors for depression included DEPRESSION IS A PROBLEM, DEPRESSION IS A LIVING ORGANISM, DEPRESSION IS A CYCLE, DEPRESSION IS BATTLE, and DEPRESSION IS JOURNEY. The use of WAR and JOURNEY metaphors were widely observed in other contexts, yet they were relatively scarce in this study. Likewise, image schemas such as SPLITTING, COUNTERFORCE, and PART-WHOLE were used to metaphorically construct these experiences, all of which are examined in the following analyses.

#### 3.1.2 People With Depression 

As shown in Table 4, participants metaphorically projected themselves as individuals with special properties, physical entities, containers, or divided entities. Their corresponding conceptual metaphors include SELF IS A SPECIAL PERSON, SELF IS A THING, SELF IS A CONTAINER, and PEOPLE WITH DEPRESSION ARE A SPLIT SELF.

**Table 4.** **Thematic fields of depression metaphors and their underlying image schemas identified from the interviews with the 20 adolescent depression patients**.

Thematic field	Source domain	Conceptual metaphor	Examples	Image schema
Depression	Thing (9)	DEPRESSION IS THING	(*1*) *a. Because in the end, what depression is **a chemical imbalance**, and all the anti-depressants do is balance the chemical back up*. [BALANCE]	COMPULSION
				PROCESS
				BALANCE
	Physical entity (8)	DEPRESSION IS PHYSICAL ENTITY	(*1*) *b. I’d say probably the media was the main thing that sort of, like all the factors probably led up to maybe me more vulnerable to depression, but the one that sort of **triggered it** and **set it off** was the media*. [COMPULSION]	COMPULSION
				PATH
				OBJECT
				VERTICALITY
				BLOCKAGE
				CONTACT
	Spatial motion (6)	DEPRESSION IS SPATIAL MOTION	(*2*) *a. I was also worried about my GCSE’s and that, and I stopped going out with my friends, and you just sort of **went downhill** from there*. [PATH]	PATH
	Location (5)			
		DEPRESSION IS LOCATION		
			(*2*) *b. Because I know we know more about depression now that we did 50 years ago, but it’s still as with most of mental health it’s still a very **vague area**.*[BLOCKAGE]	BLOACKAGE
	Container or bounded space (6)	DEPRESSION IS CONTAINER	(*3*) *a. But you don’t get any information about sort of how common it is so, but the idea that you **being alone in it **still exists.*	CONTAINER
			(*3*) *b. It’s, “I need a** release**; I need some control over something.” *[CONTAINER]	
	Problem (5)	DEPRESSION IS A PROBLEM	*I have had this for such a long time I have had the **headaches side** of it, I have had the **psychosomatic side** of depression, I have had the headaches. *[PART-WHOLE]	COLLECTION
				PROCESS
				PART-WHOLE
	Living organism (3)	DEPRESSION IS LIVING ORGANISM	*Unfortunately once I had been diagnosed, I stopped struggling with my depression and just let it** take me over***. [COMPULSION]	SPLITTING
				ENABLEMENT
				COMPULSION
	Battle (2)	DEPRESSION IS BATTLE	*I have the rest of my life ahead of me, and without the **battle** I wouldn’t have the drive to be the best I can be now. *[COUNTERFORCE]	COUNTERFORCE
	Journey (3)	DEPRESSION IS JOURNEY	*Really does but, I just thought there must, there must be some sort of meaning to this so you just got to **ride through **it and** get to the other side** I think. *[PATH]	PATH
	Cycle (2)	DEPRESSION IS CYCLE	*I’ve got so many like negative connotations to things and you just, it’s really difficult to change that, you’ve got to sort of force yourself out of the **cycle** and forcing yourself is the hardest bit. *[CYCLE]	CYCLE
Self	Individual with special properties (7)	SELF IS SPECIAL PERSON	(*4*) *a. So apart from her no-one else knew I was seeing the school counsellor because obviously I was scared of people thinking, “Oh she’s a ** nutter**.*	SUPERIMPOSITION
				NEAR-FAR
			(*4*) *b. To them I wasn’t a **victim** I was just Gemma and that made me realise that too.*	
			(*4*) *c. I wouldn’t really sort of voice my opinion too much, I mean I was, I guess I really didn’t want to be sort of like seen as **anyone just in the corner**, I’d be, I’d be fine sort of just doing my work really. *[SUPERIMPOSITION]	
	Divided entity (2)	PEOPLE WITH DEPRESSION IS SPLIT SELF	(*5*) *a. I just I want people to know that I’m** two different people**.*	SPLITTING
			(*5*) *b. But like once you get, you learn, you learn that people are just there to help you, then you will, you learn to know yourself as well, you learn to know your **new self** because like going through mental health illness it does change the person you were before into the person you become later on in life so.* [SPLITTING]	
	Physical entity (9)	SELF IS PHYSICAL ENTITY	(*6*) *a. I’m not **breaking down** at every little like hurdle. *[COMPULSION]	COMPULSION
			(*6*) *b. I’ll hyperventilate, and when I’ve **built myself up** into a panic attack I can’t think logically. *[PATH]	PATH
	Container (7)	SELF IS CONTAINER	(*6*) *c. Whereas others would be able to get round that by thinking, “Oh well this, this is fine, and I can still go out”, whereas I’d sort of like **hibernate** in my room or at home where I knew it was a safe environment. *[CONTAINER]	CONTAINER
			(*7*) *a. It’s not, of course there are always going to be some people where they just want the, want the attention whatever the cost, but if someone’s honestly having such a difficult time that’s the only way they feel they can** let out** how they’re feeling, then they need help.*	
			(*7*) *b. I’ve been about three times so far, and the first time she drew some jugs, and said, now draw a line on **how full** your good mood is today.*	
			(*7*) *c. I need to **let this out**. *[CONTAINER]	
Life	Physical entity (9)	LIFE IS PHYSICAL ENTITY	(*8*) *a. I’ve got a picture, I’ve got a collage, I’ve got, it’s sort of like** a chapter of a book writing** or something like that. *[PROCESS]	PROCESS
			(*8*) *b. The smallest things that seem so simple like having a bath or a shower, became **mountains** for me. *[BLOCKAGE]	BLOCKAGE
	SPATIAL MOTION (2)	LIFE IS SPATIAL MOTION	(*9*) *a. You’ve got to accept that things are gonna be changed, it’s hard, very hard, very hard to accept that you will have **highs and lows** because they mess you around.*	VERTICALITY
			(*9*) *b. But you know once you’ve got over that all your problems are done really, just because **highs and lows** will just be an everyday thing, they’ll just be a little routine within your daily life that you won’t have to focus on so much. *[VERTICALITY]	
	Living organism (2)	LIFE IS LIVING ORGANISM	*You know? Life** lessons** and hopefully through this I can help someone from avoiding all what I’ve had to go through. *[PROCESS]	PROCESS
	Container (2)	LIFE IS CONTAINER	*I don’t care whether, whoever wants me to **be in** that for the rest of my life, I want to** get out** and do whatever. *[CONTAINER]	CONTAINER
	Abstract Concept (2)	LIFE IS ABSTRACTION	(*10*) *And you know he, we used to write, and write to one another and stuff like that, and you know and that that was incredibly **dark** you know just, receiving a letter and knowing that it was from your father in prison. *[BLOCKAGE]	CONTACT
	Journey (2)	LIFE IS JOURNEY		PATH
School and college work	Entity (9)	WORK IS PHYSICAL ENTITY	(*11*) *a. If you’re doing your college work, and you think, “This is, this is not going well, and it’s **rubbish**, it’s pointless, what am I doing?” * [ENABLEMENT]	ENABLEMENT
				CONTAINER
			(*11*) *b. If something’s going like horrifically wrong I’ll sort of like, I’ll** dive into** my work like. *[CONTAINER]	
	Circle (2)	CAUSES AND EFFECTS ARE LINKED OBJECTS	(*12*) *a. But I just got into this **cycle**, I do not like men at all.*	CYCLE
			(*12*) *b. And then I’d get kicked out because I was messing about, but I couldn’t understand the work, so, it was just going around in **circles** really. *[CYCLE]	

The Italic texts are the metaphors that are identified from subjects’ narratives cited in the Result section. The metaphorical focus is boldfaced to indicate the verified metaphorical expressions.

In SPECIAL PERSON metaphors, adolescents with depression endowed themselves with particular properties, assuming new identities such as “a nutter”, “a victim”, or “someone in the corner” (4). This self-projection process was primarily structured by SUPERIMPOSITION schema, which involves overlaying a feature onto a target. This schema describes a conceptual process where two initially distinct entities become superimposed or merged [13]. In the following examples, participants described the properties as changes that occurred to their body after developing depression. Consequently, the self with those new properties, i.e., “nutter”, “victim”, and “someone in the corner” were understood as an imposed identity layered over their original self. Like SUPERIMPOSITION, SPLITTING schema represents the division of a single entity into two separate parts [13]. In SPLIT SELF metaphors, participants described themselves as two coexisting selves within a single body (5).

Multiple THING metaphors were employed by participants when describing themselves. Through these conceptual metaphors, individuals with depression were framed as “machine”, “building”, “bear” (6). Drawing on COMPULSION schema (6a), the “hurdles” the speaker encountered were mapped onto a compulsive external force inflicting upon her, who was framed as a machine, ultimately causing her to “break down”. When the self was likened to a building (6b), PATH schema was reflected in the metaphor: the self was understood as the source, the construction process of the “panic attack” as the path, and the “panic attack” as the goal. Along this construction path, the speaker achieved the goal by metaphorically transforming into the panic attack itself. Moreover, the self was reified as a bear (6c), aligning with the conceptual metaphor PEOPLE ARE ANIMALS. In this instance, participants utilized CONTAINER schema, which involves protection from external forces [8], highlighting the positive aspect of the undergoing containment and restraint.

Another set of CONTAINER metaphors emerged in this thematic field, where the self was objectified as a container for feelings, mood, depression (7). CONTAINER schema structured these metaphors, wherein the body was understood as a vessel, a jug or container holding moods, feelings, the disorder, or other objects within it.

#### 3.1.3 Life with Depression

When articulating their experiences of living with depression, participants discussed not only their daily routines but also specific aspects of life, such as simple tasks, employment, and personal successes or failures. These elements were projected onto various objects via conceptual metaphors. THING metaphors were prevalent in this category, shaping participants’ understanding of life with depression. Through these metaphors, their experiences were framed as a “book”, while simple daily activities were conceptualized as “a mountain” (8). A cluster of image schemas, including PROCESS and BLOCKAGE, played a role in structuring these narratives. In example (8a), the speaker mapped her life with depression onto writing a book, where her achievements were represented as chapters. In this metaphor, writing constituted a series of actions, and each completed chapter symbolized milestones within PROCESS schema, which captures the sequential nature of lived experiences. While in the narrative of (8b),BLOCKAGE schema operated in the arrangement of the speaker’s experience. Everyday experiences were metaphorically conceptualized as mountains, with the speaker herself depicted as a force encountering these obstacles that impeded her actions.

Other metaphors for life with depression included LIFE IS A LIVING ORGANISM, LIFE IS A CONTAINER, LIFE IS SPATIAL MOTION, and LIFE IS A JOURNEY, where PATH, PROCESS, and CONTAINER schemas were instrumental in structuring participants’ descriptions of their experiences.

VERTICALITY schema was prominent in metaphors related to success and failure (9), where success was conceptualized as upward movement and failure as downward descent. This image schema is fundamentally rooted in human sensorimotor experiences, imagery, and perceptual understanding of vertical motion. In this context, the speaker demonstrated a cognitive tendency to associate life outcomes with spatial orientation, equating higher positions with success and lower positions with failure. Additionally, the mapping of life onto darkness represented the CONTACT schema, in which darkness was understood as a cover over the speaker’s life and brought them into contact (10).

A set of metaphors emerged in participants’ narratives, reflecting how their academic experiences were intertwined with depression. Given that the participants were aged 15–19 and predominantly still in school, their descriptions of schoolwork and academic performance frequently conveyed negative connotations and emotional distress. ENTITY and CIRCLE metaphors frequently appeared in discussions about school tasks and college assignments, underscoring the perceived burden of academic responsibilities and their role in exacerbating depressive symptoms. ENTITY metaphors were particularly prevalent when participants described their academic work and performance in school or college, which were metaphorically conceptualized as “rubbish” and “fluid” (11), among other representations. Various image schemas were employed to structure these experiences, including ENABLEMENT and MATCHING, as illustrated in the following examples. ENABLEMENT schema captures the perceived presence or absence of power required to perform an action [8]. In this context, the speaker felt powerless to achieve success in her academic work. By utilizing ENABLEMENT schema, she mapped the process of completing college assignments onto the act of producing “rubbish” (11a), thereby highlighting the interplay between her mental health and academic performance. Conversely, when school work was compared to “fluid” (11b), CONTAINER image schema was activated. This schema accounts for the case to show avoidance of the patient’s “horribly wrong life”. The speaker conceptualized her work as a fluid substance that insulated her from the distressing aspects of her life. This perspective illustrates how containment experiences can be perceived both as constraints and as sources of security.

The following examples exemplify the conceptual metaphor CAUSES AND EFFECTS ARE LINKED OBJECTS. Their source domain of “cycle” or “circle” was derived from the speakers’ experiential patterns, structured by the CYCLE image schema. This schema represents the cyclic process that the individual begins at an initial state, progresses through a sequence of linked events, returns to the starting point, and repeats the cycle. In the first scenario (12a), the speaker expressed distrust toward male teachers in school, which triggered a sequence of causal events that perpetuated a vicious cycle in her narration. The second speaker interpreted the concept of “cycle” to illustrate the repetitive link between the difficulties she encountered in school and their eventual outcome, being “kicked out”. This metaphor underscored the iterative nature of the impact the disorder had on patients’ normal routine (12b).

### 3.2 Image Schematic Analysis of Metaphors of Moods, Thoughts and Emotions 

This section examines metaphors and image schemas associated with mood in general, specific emotions, and thought processes. As illustrated in Table 5, VERTICALITY emerged as the most frequently applied image schema in this category.

**Table 5.** **Other thematic fields of conceptual metaphors and their underlying image schemas identified from the interviews with the 20 adolescent depression patients**.

Thematic field	Source domain	Conceptual metaphor	Examples	Image schema
Moods, thoughts and emotions	Vertical position (15)	HAPPINESS IS UP/UNHAPPINESS IS DOWN	(*13*) *a. But I don’t know, when I’m on a hyper, when I’m on **a high**, I just think of so many good things, but then it just doesn’t last and then I think there’s nothing else I can do.*	VERTICALITY
			(*13*) *b. When I was at my really **low point** I danced five days a week, all night, just because I knew that when I was dancing I didn’t have those thoughts and I was happy. *[VERTICALITY]	
	Physical entity (12)	MOOD IS PHYSICAL ENTITY	(*14*) *a. Now draw a line and show us **how full** your good mood is on an average day, and how full your bad mood is on an average day.*	FULL-EMPTY
				COMPULSION
		EMOTION IS PHYSICAL ENTITY	(*14*) *b. And she was winding me up so much I was like, “The **jugs not big enough** love, it’s really not for my bad mood.” *[FULL-EMPTY]	BLOCKAGE
				COUNTERFORCE
		THOUGHT IS PHYSICAL ENTITY	(*15*) *a. And they *[*antidepressants] just didn’t, they weren’t helping, and I was like, “Yes I feel happier, but these thoughts are making me feel worse”. So it was just like a **constant conflict** between the two, and the side effects were so bad for me that I was just like, I feel sick every morning. *[COUNTERFORCE]	ENABLEMENT
			(*15*) *b. If I’d all, because sometimes my panic attacks just **jump out of** nowhere, and if it happened like that, then not a chance. *[ENABLEMENT]	
	Container (5)	EMOTION IS CONTAINER	*I thought no-one understood how I felt and finding someone that knew that made me feel happier I, that I could sort of like **open my sort of like feelings up** and say I’ve had a really shit day. *[CONTAINER]	CONTAINER
		THOUGHT IS CONTAINER		
	Thing (5)	EMOTION IS THING	*If I’d all, because sometimes my panic attacks just **jump** out of nowhere, and if it happened like that, then not a chance. *[COMPULSION]	COMPULSION
		THOUGHT IS THING		ENABLEMENT
	Living organism (3)	MOOD IS LIVING ORGANISM	*You’d have thought that I had bi-polar or something the way my moods were **flailing about**. *[ENABLEMENT]	ENABLEMENT
				COUNTERFORCE
		THOUGHT IS LIVING ORGANISM		
Communication and personal environment	Abstract concept (10)	PEOPLE ARE ABSTRACTION	*Looking back depression made my true friends **shine out**. *[REMOVAL OF RESTRAINT]	CONTACT
				REMOVAL OF RESTRAINT
	Physical entity (9)	SELF IS PHYSICAL ENTITY	(*16*) *a. I didn’t feel that I deserved their friendship, they were all very nice people, and because of the way that I had a view about myself I thought well I’m **bringing the group down**, I’m sort of depressing them. *[COMPULSION]	COMPULSION
			(*16*) *b. And, she, she was a **great support** to me at the time, and she called social services a couple of times because things at home obviously weren’t great. And yeah, she was brilliant. *[ENABLEMENT]	ENABLEMENT
	Container (9)	SELF IS CONTAINER	(*16*) *c. I’ve always been quite a quiet child, because my sister’s quite a talkative child, cos she’s quite intelligent, it sort of covered it, so I could **hide behind** her talkativeness, and, cos we went to the same schools and things together. *[CONTAINER]	CONTAINER
	Individual with special properties (4)	SELF IS SPECIAL PERSON	*But he just calls me a **nutter**. *[SUPERIMPOSITION]	SUPERIMPOSITION
	Thing (3)	IGNORANCE IS THING	(*16*) *d. And that automatically creates **friction** within a family environment. *[CONTACT]	CONTACT
				REMOVAL OF RESTRAINT
		TRUST IS THING	(*16*) *e. It’s getting over that **hurdle** of trusting them is the hardest thing with the relationship you just break down the trust, there’s no trust, I mean I trust people now but it’s, it’s still difficult to truly believe that they’re not gonna hurt you. *[REMOVAL OF RESTRAINT]	
		FRIENDSHIP IS THING		
	Living organism (2)	ARGUMENT IS LIVING ORGANISM	(*16*) *f. I ignore him most of the time but my mum and dad are always screaming at each other which is very difficult to **live with**. *[BLOCKAGE]	BLOCKAGE
Medicine and therapy	Living organism (8)	MEDICINE IS LIVING ORGANISM	(*17*) *a. Although having said that medic students do drink a hell of a lot, so, they are, they obviously **don’t listen to** their own medicine, so to speak. *[DIVISION]	DIVISION
				COMPULSION
		COUNSELLING IS LIVING ORGANISM		CONTACT
			(*17*) *b. They’ve gave me some paranoia and anxiety tablets, when I, but I never took. I don’t take, I was scared of what they’re gonna **do** to me. * [COMPULSION]	
	Physical entity (6)	THERAPY IS PHYSICAL ENTITY	(*18*) *a. But with depression there is, as I said there is no **plaster**, there is no, there is no, obvious very simple thing. *[LINK]	LINK
				BLOCKAGE
		PATIENT IS PHYSICAL ENTITY	(*18*) *b. It’s a **process** and they’re one of the **steps** in the **process**, I don’t see anything wrong with them now. *[PROCESS]	ITERATION
			(*19*) *At first it’s like a **chore**, because you’ve been, it’s like you’ve been given a task to do and you’ve got to do it, and it feels like a chore to carry on doing it, and being able to apply it all the time. *[ITERATION]	
	Container (5)	THERAPY IS CONTAINER	(*20*) *a. Waiting to go in into the doctors to say, can I have a referral, or therapy or, or even going to your first therapy session, you’re **stood out-side** you know, you know you stand and chain smoking.* [CONTAINER]	CONTAINER
		COUNSELLING IS CONTAINER		
			(*20*) *b. Then you **get in** and when you’re with the people you start to*	
			*feel more relaxed, you felt more like you were just like talking with your mates like you just felt like you were the same. *[CONTAINER]	
Habits and interests	Location (4)	HABIT IS LOCATION	(*21*) *a. I go to the gym a lot, and it just **gets you away from** thinking of different thoughts, and socially it’s really good cos you meet other people that have got the same sort of interest as you, like my dancing, it **got me away from** my thoughts.*	PATH
			(*21*) *b. It’s making me a million times worse, I’m not **coming back down** that road, I’m not going, you know. *[PATH]	
	Container (2)	HABIT IS CONTAINER	(*22*) * But at the minute I’m just trying to **get out** of that habit and just start being back to the way I was again. *[CONTAINER]	CONTAINER
	Abstract concept (2)	HABIT IS ABSTRACTION	(*23*) *I used to like skip meals and see if I could just, and every day I went without eating it was like a **bonus** for me, it was like, it was like a **pat on the back** to myself. *[ATTRACTION]	ATTRACTION
Others	Physical entity (10)		(*24*) *a. From like the point of like 11 on, up until I was about 14 I just basically **got on** with my day when I was feeling depressed and stuff. * [ENABLEMENT]	SCALE
				ENABLEMENT
				OBJECT
			(*24*) *b. And something like that *(*bullying*) *it does **scar** you for life, people don’t see it, but in mentally and physically **scars** you for life. *[BLOCKAGE]	BLOCKAGE
			(*24*) *c. With the help of my brilliant counsellor slowly my confidence **built up** and I started living again by joining a young advisory group and going to short college courses. *[SCALE]	
			(*25*) *a. Cos obviously it’s a **scar** for life really. *[OBJECT]	
	Abstract concept (4)		(*25*) *b. I mean the self-harm thing went on for a good couple of years and it’s, you know, like smoking it’s an **addiction**, and just saying, “Right I want it to stop now, and that’s it.” *[ATTRACTION]	ATTRACTION
	Place (4)		(*26*) *Or just put it to the **back of my mind** so I didn’t get, look upset no more. *[PATH]	PATH
				CONTAINER

The Italic texts are the metaphors that are identified from subjects’ narratives cited in the Result section. The metaphorical focus is boldfaced to indicate the verified metaphorical expressions.

The analysis showed a correlation between mood metaphor and the types of image schema. It was observed that when describing their moods, subjects tended to use conceptual metaphor HAPPINESS IS UP to conceptualize positive emotions and UNHAPPINESS IS DOWN to convey negative emotional states. Consistent with previous works [15], the analysis showed that VERTICALITY image schema was overused in the construction of these experiences. As shown in example (13), speakers perceived mood in a perpendicular direction, and projected good mood with an elevated position and bad mood with a downward position. In addition to VERTICALITY, another type of metaphor was structured around the FULL-EMPTY schema, which is the abstracted structure of “emptiness” and “fullness” based on physical experiences. For example, when a cup overflows with water, we perceive it as full; when half of the liquid is spilled, we consider it half-full; and when the water is completely consumed, we describe it as empty—all based on prior sensory experiences. Here, the metaphor conceptualized mood as a fluid contained within a jug, and the speakers’ mood was quantified by drawing a line to show “how full” it was (14).

However, no relativity was identified between a particular image schema and the frame of metaphors concerning a specific thought or emotion. Thoughts and emotions were variously framed as physical objects, general entities, animate beings, or containers, suggesting that a range of image schemas played a role in organizing these experiences. Among them, FORCE-related schemas such as COUNTERFORCE and ENABLEMENT (15) were particularly relevant. Drawing on the cognition structure of COUNTERFORCE schema, which involves the direct opposition of forces, the subject structured the perception of thoughts and happiness as colliding forces engaged in a head-on encounter, producing an ongoing state of conflict (15a). This may have caused adverse impacts on the mental health of the participant. In example (15b), panic attacks were conceptualized as animate things. Here, ENABLEMENT schema exhibited its function differently, shaping the reasoning behind the speaker’s articulation. It represented panic as an active force capable of initiating an action, such as “jumping” within the given context. This suggests that the ENABLEMENT schema is not only applicable to human agency, where the speaker perceives themselves as possessing the ability to act, but also extends to non-human entities, attributing them with animate characteristics.

### 3.3 Image Schematic Analysis of Metaphors of Communication and Personal Environment

People with depression often experience difficulties in their social activities [17]. The target domains of metaphors regarding communication and personal environment comprised interrelationships, social environments, conversations, as well as particular people in the social context. As shown in Table 5, CONTAINER, ENABLEMENT, and COMPULSION were commonly employed in the construction of these metaphors.

Metaphors framed through CONTAINER, PHYSICAL ENTITY, ABSTRACTION, and LIVING ORGANISM schemas were particularly prominent, with FORCE-related schemas playing a significant role in this thematic field. For instance, COMPULSION schema was utilized in example (16a), where the speaker perceived herself as a destructive force capable of damaging friendships. In (16b), ENABLEMENT schema shaped the metaphorical representation of the teacher as an external source of support, enabling the speaker to navigate life’s challenges. Regarding the family environment (16f), BLOCKAGE schema structured the speaker’s perception of parental conflicts as barriers impeding normal interactions. Another FORCE image schema, REMOVAL OF RESTRAINT, functioned in organizing the speaker’s experience of intimate communication with others (16e), where trust was metaphorically framed as an obstacle hindering intimacy between the speaker and her friends. This schema is characterized by the elimination of a restraining barrier, thereby opening a path that allows force to be exerted. In this case, overcoming trust issues was equated with removing barriers to facilitate deeper communication. CONTACT schema was present in example (16d), where a lack of communication was depicted as friction, leading to strained interactions between the speaker and her family. Finally, in (16c), CONTAINER schema was used to represent the speaker’s sister’s talkativeness as a protective enclosure, shielding the speaker from potential harm.

### 3.4 Image Schematic Analysis of Metaphors of Medicine and Therapy

During the interviews, participants employed a subset of metaphors to describe the medication, counseling, and treatments they had undergone. LIVING ORGANISM metaphors were particularly prominent in conceptualizing medicine and pills, as they were attributed with agency and the capacity to perform specific actions. FORCE-related image schemas were frequently utilized in reasoning these conceptual metaphors. The examples below objectified medicine as a physical force, with people either positioned as its target or as an opposing force engaging in confrontation. This clash metaphorically represented the resistance between individuals and their prescribed treatment, as in the expression “people don’t listen to their medicine (17a)”. This metaphor exemplified the DIVISION schema, which characterizes the collision of two opposing forces, resulting in a change in their respective trajectories. While in the illustration of COMPULSION schema, the owner of the medicine was conceptualized as the target on which the powerful force of medicine was about to exert, and it would somehow yield negative impact on the target (17b).

Regarding metaphors for counseling and treatment, the source domains included physical entities, locations, bounded spaces, which are structured by COMPULSION, PROCESS, and LINK schemas. Depression was metaphorically framed as a physical wound, with its treatment conceptualized as a plaster covering the injury (18a). This aligns with the conceptual metaphor PSYCHOLOGICAL HARM IS PHYSICAL INJURY, which is supported by the LINK schema, rooted in human perception of similarity [8]. This metaphor reflected the speaker’s skepticism toward medication for depression, as expressed in the sentiment “no plaster could be used for this disorder”. Conversely, example (18b) demonstrated a contrasting perspective, where the speaker maintained a more positive outlook on medication and treatment for depression. Guided by the cognitive structure of PROCESS schema, the treatment of depression was conceptualized as an ongoing process, with medication representing one of its integral steps. In discussing their treatment experiences, participants also employed ITERATION schema to structure their perceptions of self-directed efforts in managing depression. For instance, maintaining a positive mindset to counter depression was metaphorically framed as a chore (19), a routine task that the speaker felt compelled to sustain over an extended period. Moreover, CONTAINER schema was utilized by participants to describe therapy and treatment. In this case, therapy sessions and counseling were metaphorically depicted as enclosed spaces, specifically designated for individuals requiring support (20).

### 3.5 Image Schematic Analysis of Metaphors of Habits and Interests

This study also examined a subset of metaphors related to participants’ personal hobbies, interests, and daily routines, structured by LOCATION, CONTAINER, and ABSTRACTION schemas, as these may provide valuable insights into adolescents’ lived experiences with depression. Drawing on PATH schema, example (21a) illustrates how the speaker conceptualized their interests in exercising and dancing as animate entities, while the act of engaging in various thoughts was framed as a location. In this context, personal interests were perceived as a positive force in managing depression. Conversely, in example (21b), a habit the participant developed after experiencing depression was also metaphorically construed as a location. However, as this habit was harmful to the speaker’s health, they expressed reluctance to return to it.

A particularly noteworthy case (23) highlighted a contradiction between the speaker’s perception of a habit and its actual impact. Here, the habitual practice of skipping meals was metaphorically framed as a “bonus”, something the speaker regarded as a reward or encouragement, reinforcing their motivation to continue the behavior. This metaphor exemplified ATTRACTION schema, as the speaker was drawn to this habit due to the immediate gratification it provided. However, in reality, this practice was detrimental to their physical well-being. CONTAINER schema also played a role in structuring metaphors related to changes in personal routines. In example (22), the speaker conceptualized a present habit, altered by the onset of depression, as a container from which they were attempting to escape. These metaphors revealed a dual perspective on personal interests and habits in relation to depression. On one hand, engaging in hobbies and interests could serve as a coping mechanism. On the other, newly developed habits following the onset of depression were more damaging to adolescents’ health.

### 3.6 Other Metaphors

Finally, a set of metaphors emerged in relation to the mind and brain, self-harm, time, bullying, personal beliefs, and individual characteristics. These conceptual metaphors were categorized under PHYSICAL ENTITY, LOCATION, LIVING ORGANISM, and ABSTRACTION metaphors. ENABLEMENT, OBJECT, PATH, SCALE, BLOCKAGE, and ATTRACTION schemas provided the inferential structures supporting the source-target domain mappings in the following examples.

In example (24a), the speaker described their experience of depression in terms of a path they were traveling along. Utilizing ENABLEMENT schema, the phrase “got on” implied that the speaker was able to continue moving forward along a time path spanning approximately three years. Example (24b) illustrated metaphors related to bullying. In this context, the speaker described being left with a lasting psychological scar after experiencing bullying—a wound invisible to others but exerting a lifelong influence on them. Framed through BLOCKAGE schema, this metaphor depicted the lingering impact of bullying as a mental scar obstructing the speaker’s ability to move forward in life. During the interviews, participants also reflected on specific personal attributes they associated with their experience of depression. In line with the conceptual metaphor MORE IS UP, the increase in confidence was metaphorically represented as upward movement (24c). The construction of such metaphors was motivated by SCALE schema, which pertains to quantitative variations in human experiences, such as the increase or decrease of a given attribute. Several metaphors emerged concerning the mind and brain. In example (26), the mind or brain was conceptualized as a bounded space, delineated by clear mental boundaries. Within PATH schema, the process of overcoming sadness was metaphorically described as moving this emotion along a path to an endpoint—the “back part” of the speaker’s mind in this case.

Beyond external influences negatively affecting adolescents diagnosed with depression, some participants referred to tendencies or direct experiences of self-harm. Several metaphors associated with self-harm emerged in their narratives, conceptualizing it as either a permanent scar or an addiction (25). The former conceptualization aligned with OBJECT schema, which attributes physical properties to abstract concepts. The latter was linked to ATTRACTION schema, reflecting the compulsive pull of self-harming behaviors. Despite different experiential structures, both metaphorical representations underscored the persistent negative interplay between depression and self-harm, highlighting the difficulty adolescents faced in disengaging from such behaviors. The domain mappings identified in these examples emphasized the enduring detrimental effects of bullying and self-harm on participants. While the frequency of metaphors addressing these themes was relatively low, the analysis suggests that self-harm and bullying significantly lead to the worsening of both the mental and physical health conditions of adolescents experiencing depression.

## 4. Discussion

This study conducted an in-depth analysis of metaphors in the narratives of adolescents with depression. The findings not only corroborated the conceptual metaphors identified in previous studies, but also revealed new metaphors specifically used to describe the multifaceted nature of depression among adolescents. These metaphors collectively reflected the concerns of adolescents suffering from depression. Furthermore, the interpretation of metaphors through Image Schema Theory provided insights into adolescents’ metaphorical thinking processes and the source-target domain mappings of their depression experiences. This, in turn, shed light on the cognitive framing mechanisms of depression.

Firstly, this study found that 40.82% of metaphorical expressions were associated with depression. However, these expressions did not pertain solely to the disorder itself, but also extended to individuals diagnosed with depression, their daily lives, and academic responsibilities. Consistent with prior research, CONTAINER metaphors were extensively used in participants’ narratives related to depression. In most cases, depression was conceptualized either as a container or as a bounded space that restricted individuals, or the individuals themselves were depicted as containers holding negative emotions [14]. Furthermore, this study observed four novel types of container metaphors: (1) life with depression was conceptualized as a container that oppressed individuals, prompting a desire to escape; (2) therapies were framed as containers where treatment activities took place, reinforcing the perception that patients were separated from healthy individuals; (3) habits were depicted as containers, reflecting the persistent negative influence of depression; and (4) interpersonal attributes were understood as containers that shielded individuals with depression from external harm.

These findings further validated that individuals with depression were often framed in terms of containment and restriction [14]. Moreover, patients appeared to be introducing new types of containers as a means of self-isolation [17]. Beyond the limitation of agency, this study also revealed an alternative function of the container metaphor, serving as a protective barrier that shielded adolescents with depression from external disturbances. This illustrated how adolescents’ sense of limitation was projected onto the container metaphor, not only as a form of restriction but also as a defense mechanism against external pressures.

Furthermore, this study identified metaphors portraying adolescents with depression as individuals with dual identities—either as a divided entity or as a unified self-endowed with a new identity. Such metaphors have been recognized in a previous study and categorized as SPLIT SELF metaphors [34]. The interpretation of image schemas in the present study aligned with Qiu *et al*. [13], who found that trauma patients exhibiting dissociation symptoms frequently employed SPLITTING and SUPERIMPOSITION schemas to structure their lived experiences. Similarly, this study observed that a sense of dissociation also occurred in the context of depression. Participants tended to perceive their original, pre-depression selves as “normal”, while regarding their depressed selves as a distinct new identity. In SPLITTING schema, patients were metaphorically depicted as a body containing two separate identities. In contrast, SUPERIMPOSITION schema framed depression as a new identity layered over the individual’s original self.

Unlike adults, adolescents encounter unique challenges, such as school transitions and academic failure, and metaphors can facilitate the interpretation of these struggles [11]. Given that participants in this study were still in school or college, they often framed their academic workload as burdensome entities, such as “mountain” and “rubbish”. This suggested that depression negatively impacted adolescents’ well-being and that academic-related stress contributed to their distress. Consequently, healthcare professionals should pay closer attention to academic pressures as potential risk factors for adolescents with depression. Additionally, numerous metaphors were used to express negative emotions, moods, and thoughts among adolescents suffering from depression. According to Yu and Tay [15], VERTICAL ORIENTATION metaphors frequently occur when individuals describe their moods, often in terms of being “low” or “down”. Consistent with this observation, the present study found that participants metaphorized mood fluctuations along a vertical axis, drawing upon experiential patterns derived from VERTICALITY image schema. Furthermore, this study identified metaphors that conceptualized moods as fluid contained within a jug, instantiating FULL-EMPTY schema, which reflected variations in emotional intensity. Other conceptual metaphors mapped specific emotions and thoughts onto physical entities, particularly nuisance-related objects, and were structured by OBJECT, CYCLE, COUTERFORCE, and ENABLEMENT schemas.

In addition, adolescents with depression in this study placed significant emphasis on their social environments, as evidenced by the frequent use of metaphors describing interpersonal relationships and social settings. Consistent with Coll-Florit *et al*. [17], social communication was primarily framed in terms of restriction and limitation. These metaphors depicted social interactions through FORCE-related image schemas, including COMPULSION, ENABLEMENT, and BLOCKAGE, which either represented barriers that hindered interpersonal relationships or the removal of such obstacles to facilitate communication. Notably, ENABLEMENT schema assumed a new role in expressing empowerment, as it was used to articulate how individuals gained social support through interpersonal connections [35]. This study suggested that adolescents metaphorically transformed external support into personal resources, enabling them to take actions to overcome depression. In line with McMullen [36], the findings highlighted that depression exposed the ways in which individuals were either isolated or supported by society.

Furthermore, metaphors relating to medicine and therapy were mapped onto living organisms, containers, and various physical entities. These metaphorical projections primarily drew upon CONTAINER, COMPULSION, and PROCESS schemas. However, this study did not observe metaphors depicting medical treatment as stripping patients of their agency, as proposed by Coll-Florit *et al*. [17]. Instead, metaphors of medical practices primarily reflected patients’ perceptions and attitudes toward treatment outcomes.

This study also expanded the scope of metaphors concerning patients’ depression experiences to habits, interests, bullying, and self-harm. For example, habits and interests were frequently conceptualized as places or locations, with PATH schema framing these experiences as movements toward or away from specific locations. Given that self-harm and suicidal behaviors are particularly prevalent among depressed adolescents [37], Bennett *et al*. [37] argued that young individuals often construct depression as a disease that limits their agency within medicalized discourse. This parallels Sasala, Mudogo, and Barasa’s findings [38], which demonstrated that the normalization of COVID-19 through metaphors hindered public health efforts to curb its spread. Likewise, adolescents who viewed self-harm as an ordinary and acceptable behavior were at greater risk of worsening their mental and physical health. This underscores the need for healthcare professionals and caregivers to engage in more frequent discussions with adolescents to correct misconceptions and improve their understanding of harmful behaviors.

### 4.1 Limitations and Future Research

This study has several limitations. First, the sample size is relatively small. This study conducted a qualitative analysis of depression metaphors produced by 20 adolescent patients, which limits the generalizability of its findings. The second limitation concerns metaphor interpretation. The source domains of metaphors were classified into broad categories and analyzed collectively. Only the most salient and distinctive cases were highlighted, which may have led to the omission of some vivid mental imagery describing patients’ lived experiences with a mental disorder. Regarding the analysis of image schemas, although correlations between certain metaphorical themes and image schemas were explored, further research is needed to deepen the understanding of the clinical implications of image schematic metaphors. Moreover, since this study employed a qualitative methodology, the analysis focused primarily on general discourse patterns concerning the prevalence of conceptual metaphors and image schemas. However, it did not compare the proportions of metaphorical versus literal content across individual topics, which represents a limitation in the study’s comprehensiveness.

These limitations suggest that future research should expand the sample size by including a broader range of interviews to validate these findings within a larger population of individuals with depression. A more extensive qualitative analysis of conceptual metaphors would provide deeper insights into adolescents’ interpretations of their experiences with depression. Furthermore, a detailed comparison between the proportion of metaphorical and literal content across different topics and within individual interviews could enhance the precision of future analyses. Lastly, follow-up studies could incorporate statistical models to quantitatively examine and verify correlations between specific image schemas and metaphorical themes in the context of depression.

### 4.2 Implications for Practice

The findings of this study supported the use of conceptual metaphors as an essential communication tool, demonstrating their significant value in improving the understanding of health-condition discourse, particularly among adolescents with depression. Additionally, the analysis of image schemas underlying these conceptual metaphors provided insights into adolescents’ metaphorical thinking processes and the source-target domain mappings in their depression narratives. Thus, the present study shed light on the application of image schemas and conceptual metaphors to managing health issues and facilitating psychotherapy for adolescent patients with depression.

In practice, a bodily perspective on metaphor, specifically, an image-schema-based metaphor strategy, could contribute to therapeutic interventions [39]. The core concept is to integrate the body as both a focal point and a resource in therapy, facilitating an overlap between metaphor, bodily experience, and psychotherapy. By employing the “problem-solution framework”, in which problems can be addressed through concrete physical actions [40], therapists can reinterpret clients’ mental health challenges through metaphors rooted in embodied experiences. Metaphors can serve as a bridge, facilitating a transition from conceptual, body-based representation to tangible, body-based intervention.

In Tay’s reported metaphor-body-psychotherapy extract 3 [39], the client used two metaphors to describe his feelings of shame and guilt, as the way others see him was conceptualized as “branding his face with a ‘homosexual’ stamp”, and his living with shame as “walking aimlessly in a dead town, not knowing if there’s a cliff or anything else ahead”. During psychotherapy, the therapist reconstructed the client’s shame as “a physical substance” using the embodied CONTAINER metaphor, in which shame was envisioned as being hidden in the client’s abdomen and capable of being released through verbal expression. Drawing on the embodied experience of containment, the therapist encouraged the client to verbalize and externalize his feelings of shame by saying “I feel ashamed”, instructing him to “let the abdomen speak it out”, and guiding him to physically touch his abdomen. In doing so, the client actively confronted his shame and experienced “shame loss” through these instructed behaviors. This step facilitated a convergence between conceptual reasoning (target) and physical action (source), engaging the client in both the source domain scenario, removing a substance from his abdomen, and the target domain scenario, processing and verbalizing his shame. In the present study, a participant made the following statement while discussing depression: “Unfortunately once I had been diagnosed, I stopped struggling with my depression and just let it take me over”. In this metaphor, the participant construed depression as an external force that had the patient at its command. This metaphor drew upon the experiential pattern of COMPULSION schema, as the patient perceived an overwhelming force acting upon them, leading to an uncontrollable outcome. In this case, the patient emphasized their sense of involuntariness and vulnerability, highlighting how easily they felt overtaken by the illness. Now, if this statement were made by a patient during therapy, the therapist could implement the image-schema-based metaphor strategy discussed earlier in subsequent sessions. The first step would involve reconstructing the client’s depression as a tangible physical entity, such as “a big rock blocking their path”, “a troublemaker”, “a substance inside their body”, or another concrete metaphorical representation. This approach helps the client conceptualize their problem as something that can be actively addressed through physical action. The next step would be guiding the client to “manage” their problem. The therapist could employ ENABLEMENT schema to encourage the client to reclaim agency and power by “accessing” sources of strength from their environment—for example, sunlight, engaging activities, family, and friends. Through concrete actions, the client could be encouraged to “remove the big rock from their path”, “confront the troublemaker”, or “expel the substance from their body”. The key is to redirect the client from COMPULSION to ENABLEMENT, to transform her from passively accepting depression to actively collecting power and acquiring agency so as to deal with depression via image-schema based metaphor strategy.

Similarly, if patients metaphorically projected their concerns and challenges as obstacles or barriers, therapists could engage them in conceptualizing these barriers both cognitively and physically through image-schema-based metaphors. These obstacles could be made accessible through body-based activities and behaviors. By applying the REMOVAL OF RESTRAINT schema, therapists could guide patients to physically interact with the metaphoric obstacles blocking their path. As patients push these obstacles aside and free themselves from hindrances, they simultaneously develop a tangible, embodied experience of confronting and eliminating negative thoughts and concerns from their minds.

## 5. Conclusions 

This study examined the metaphorical expressions produced by adolescents with depression, along with the various image schemas that structured these metaphors. The analysis provided insight into adolescents’ experiences of depression, highlighting that metaphors serve as essential linguistic resources that patients use to articulate their experiences with the disorder. In addition, the findings demonstrated how adolescents’ cognitive framing mechanisms operate and how target-source domain mappings are established. It can be concluded that adolescents’ conceptualization of complex emotions and fragmented experiences through metaphors relies heavily on fundamental experiential patterns derived from image schemas.

Through metaphor analysis, this study has broadened our understanding of various aspects of patients’ lives with depression, including their social environments, interpersonal relationships, treatment experiences, personal habits, bullying experiences, and self-harm. These factors presented a rather comprehensive picture of what patients were really concerned about and struggled with, and this calls on people within their social milieu to engage in more frequent and meaningful communication with them, in order to address their dynamic emotional and physical needs. Furthermore, the analyses of image schemas substantiated a correlation between certain themes and image schema. Specifically, adolescents with depression tended to communicate their moods by exploiting the recurrent patterns of VERTICALITY image schema and identity clash by SPLITTING and SUPERIMPOSITION schema. This suggests that image schemas could serve as a valuable tool for mental health professionals in assessing individuals’ mental health status before implementing conventional screening measures.

The findings about image schemas could also offer practical clinical implications for counselors and therapists, particularly in employing experiential patterns associated with empowerment and gaining agency into counseling and therapeutic interventions. For instance, counselors and therapists may use the framing structure generated from ENABLEMENT image schema, which depicts the possession of a power to overcome challenges, to help patients reframe their perception of difficulties as passively accepted norms dictated by the COMPULSION schema. Besides, medical professionals may shift patients’ thinking patterns by switching the containment and restraint motivated by CONTAINER schema to the protection and shelter it can provide for them. When patients metaphorically project their concerns onto barriers, medical professionals may draw on REMOVAL OF RESTRAINT schema to reconstruct their mental representations, encouraging them to visualize the elimination of obstacles and empowering them to progress in their recovery.

## Availability of Data and Materials

The data that support the findings of this study are available on request from the corresponding author.
